# Nanoparticle-encapsulated hepcidin as a dietary strategy to improve growth, biochemical status, and immune responses in common carp (*Cyprinus carpio*) pre- and post-*Aeromonas hydrophila* challenge

**DOI:** 10.1016/j.vas.2026.100835

**Published:** 2026-08-22

**Authors:** Ehsan Ahmadifar, Sedigheh Mohammadzadeh, Mohsen Shahriari Moghadam, Mostafa khajeh, Hossein Adineh, Sevdan Yilmaz, Najmeh Sheikhzadeh, Majid Khanzadeh

**Affiliations:** a Department of Fisheries, Faculty of Natural Resources, University of Zabol, Zabol, Iran; b Amol University of Special Modern Technologies, Amol, Iran; c Department of Environmental Sciences, Faculty of Natural Resources, University of Zabol, Zabol, Iran; d Department of Chemistry, University of Zabol, Zabol, 98615-538, Iran; e Department of Fisheries, Faculty of Agriculture and Natural Resources, Gonbad Kavous University, Gonbad Kavous, Golestan, Iran; f Çanakkale Onsekiz Mart University, Department of Aquaculture, Faculty of Marine Science and Technology, Çanakkale, 17100, Turkey; g Department of Food Hygiene and Aquatic Animals, Faculty of Veterinary Medicine, University of Tabriz, Tabriz, 51666-14766, Iran; h Animal Biological Product Research Group, Academic Center for Education, Culture and Research (ACECR), Tehran Organization, Tehran, 1314744513, Iran

**Keywords:** Common carp, Hepcidin, Nano-carboxymethyl chitosan, Nano-β-cyclodextrin, Immuno-biochemical indices

## Abstract

Various antimicrobial peptides are small peptides with potential for controlling pathogenic microorganisms. Nanoencapsulation of antimicrobial peptides is a strategy to limit some challenges, such as low stability, and improve the efficacy of these antimicrobial drugs. This study was designed to assess the efficacy of encapsulated hepcidin within nano-carboxymethyl chitosan and nano-β-cyclodextrin compared to natural hepcidin on the growth, immunity, and biochemical responses in common carp (*Cyprinus carpio*) following the challenge with *Aeromonas hydrophila*. Fish (5.05 ± 0.22 g) were acclimatized and then divided into four experimental groups supplemented with a control diet (CTR), a diet with natural hepcidin (Hep), a diet with hepcidin within nano-carboxymethyl chitosan (CMCS-Hep), and a diet with hepcidin within nano-β-cyclodextrin (βCD-Hep) for 8 weeks and then challenged with pathogenic *A. hydrophila*. Results showed that fish fed the CMCS-Hep diet demonstrated significant differences in final weight, liver-related enzyme activities, stress and immune indices compared to the CTR and Hep groups. In conclusion, the present study showed that common carp might appear to benefit from encapsulated hepcidin within nano-carboxymethyl chitosan in terms of growth, biochemical and immunological traits.

## 1 Introduction

The aquaculture industry worldwide has experienced swift and significant growth during recent years. Various types of finfish, e.g., tilapia, catfish, sea bream and carp, are the main species for aquaculture production. Common carp (*Cyprinus carpio*) is one of the widely cultivated and significant commercial freshwater fish species in the world (Mohammed et al., 2024). *Aeromonas hydrophila* is one of the most prevalent bacteria isolated from carp culture systems, with gastroenteritis, septicemia, and necrotizing fasciitis as the most prevalent signs of disease (Semwal et al., 2023).

Natural and artificial antimicrobial peptides (AMPs) have received attention during recent years thanks to their biological activities as a great approach to conventional antimicrobial components combating drug-resistant and pathogenic microorganisms. These biomolecules, as the key protective components in fish species, provide the first line of defence against microbial pathogens, including viruses, fungi, and bacteria (Fadaka et al., 2021). Fish AMPs are broadly classified into defensins, piscidins, cathelicidins, hepcidins and histone-like peptides. Fish hepcidin, as the liver-expressed antimicrobial peptide of the LEAP family, is a cysteine-rich AMP with a β-sheet structure with four disulfide bonds (Gao et al., 2024). Previous studies have proved the high expression of hepcidin in response to bacterial agents in fish species (Chen et al., 2018; Barroso et al., 2021; Lee et al., 2022; Gao et al., 2024). Despite the health effects caused by AMPs, they have some limitations such as low bioavailability and proteolytic susceptibility, delaying their progress into clinical trials (Divyashree et al., 2020; Del Genio et al., 2022). The direct use of purified synthetic peptides in aquafeeds may not always be economically practical for routine production, particularly in low-value species such as common carp. However, in the present study, highly purified hepcidin was used as a model bioactive molecule to evaluate whether nano-encapsulation could improve its functional performance and provide a basis for future development of more efficient peptide-based delivery strategies.

Cyclodextrins (CDs), as cyclic oligomers originating from starch enzymatic degradation, have the potential to establish supramolecular host–guest interactions due to their toroidal shape and non-polar interior. Because of their special architecture forming inclusion complexes with various types of molecules like ions, proteins, and oligonucleotides, these biomolecules contribute distinguished advantages. Cyclodextrin complexation represents an effective strategy for improving protein therapy by stabilizing proteins against aggregation, thermal denaturation, and degradation (Mejia-Ariza et al., 2017; Pandey, 2021). Chitosan is the most broadly used linear polysaccharide for diverse applications in different fields such as the textile industry, agriculture, cosmetics, and food processing. Regarding biocompatibility, biodegradability, and non-toxicity, this polysaccharide is considered an excipient in drug formulation. Carboxymethyl chitosan (CMCS) was chosen in this study because its water solubility, film-forming ability, and favorable interaction with bioactive molecules may improve peptide protection and release under digestive conditions. It was previously proven that lowering the size of different particles to the nanoscale could enhance their solubility, mobility, and efficacy of different materials compared to the larger ones (Khani Oushani et al., 2020). A delivery system based on nanotechnology has been considered a beneficial approach to improve the therapeutic efficacy of the AMPs via preventing proteolysis, increasing AMP accumulation at the infection sites, and reducing bystander toxicity (Del Genio et al., 2022). In parallel, AMPs encapsulation into chitosan nanoparticles (NPs) could overcome the therapeutic limitations of AMPs (Jayathilaka et al., 2022). To our knowledge, no previous study has investigated the effects of hepcidin encapsulated in nano-carriers such as CMCS and β-cyclodextrin (β-CD) on growth performance and physiological indices in common carp. Most previous studies on fish hepcidin have primarily focused on its antimicrobial activity or immune-related functions, whereas little information is available regarding the physiological consequences of nano-delivered hepcidin in aquaculture species. Therefore, this study provides a novel approach by comparing free hepcidin with nano-encapsulated hepcidin in two different delivery systems, aiming to evaluate whether nano-formulation can improve the functional efficacy of this antimicrobial peptide in common carp. Based on the above considerations, we hypothesized that CMCS and β-CD would improve the biological efficiency of hepcidin compared with its free form. Therefore, the main objective of this study was to evaluate and compare the effects of free hepcidin, nano-CMCS-hepcidin, and nano-β-CD-hepcidin on growth performance and physiological indices of common carp.

## 2 Materials and methods

### 2.1 Synthesis and nanoencapsulation of hepcidin

**Fig. 1:**
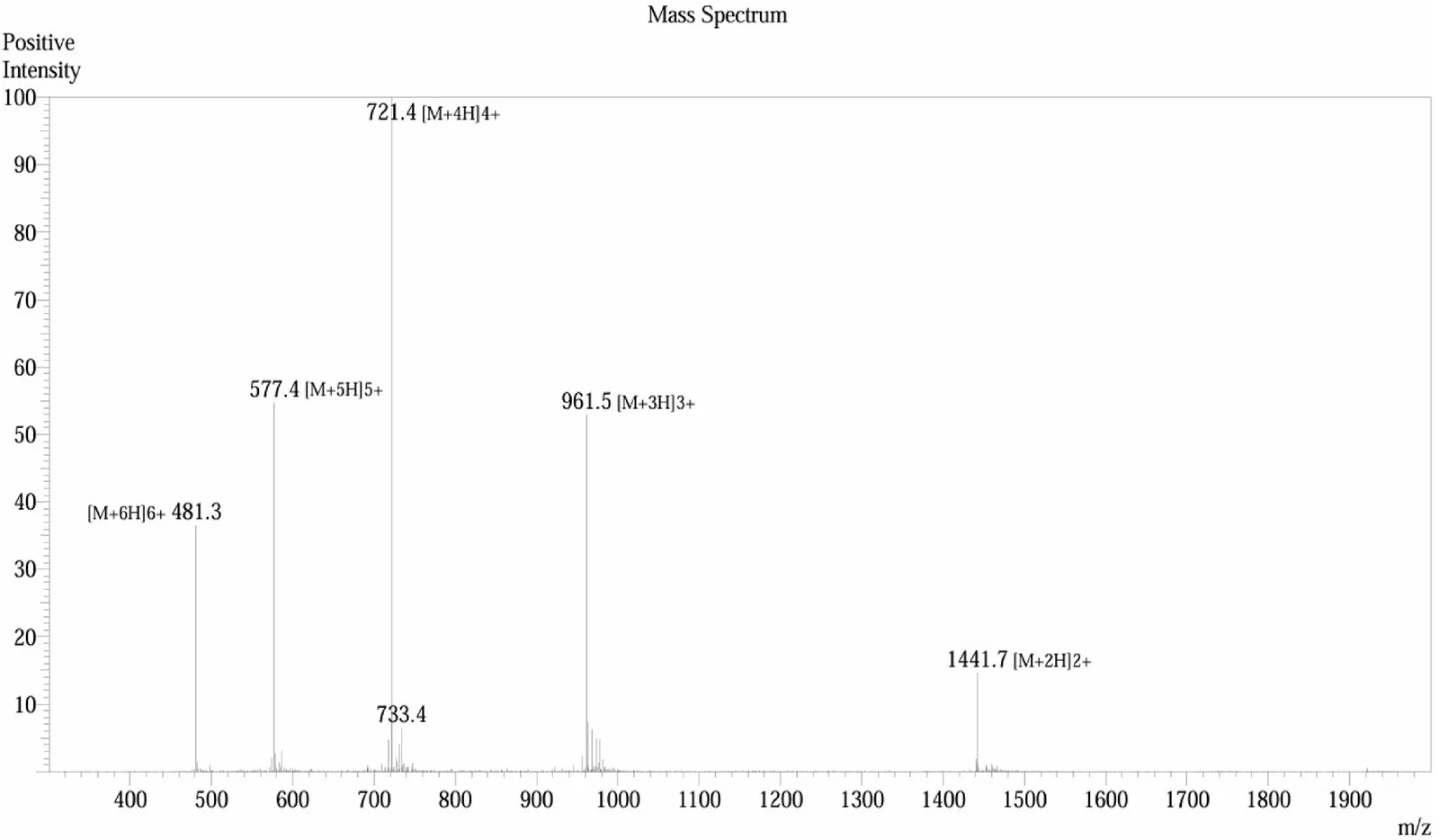
Mass spectrometry analysis of the synthetic *C. carpio* Hepcidin. The expected mass of 2881.6 Da was confirmed, indicating that all four disulfide bonds are present in the mature peptide**.**

The mature hepcidin peptide (QSHLSLCRWCCNCCHNKGCGFCCKF, at 99% purity) was synthesized (GenScript company, China), and the molecular mass (2881.6 Da) was confirmed by mass spectrometry analysis (Fig. 1). CMCS NPs were prepared via ionic gelation using CaCl₂ (5%, w/v) as a crosslinker at a 1:1 CMCS: CaCl₂ weight ratio under stirring for 1.5 h and kept for 72 h at room temperature. For hepcidin conjugation, 500 mg of CMCS NPs were suspended in acetate buffer (pH 5.5), and 1 mg of hepcidin was activated with EDC/NHS for 30 min, then added dropwise to the NP suspension. The reaction proceeded for 4 h to form amide bonds, followed by centrifugation to isolate the conjugated NPs (Wang et al., 2022; Karimzadeh et al., 2022). Similarly, β-CD was activated in dimethyl sulfoxide (DMSO) with 1,1′-carbonyldiimidazole (CDI) for 4 h, then reacted with hepcidin (in DMSO) for 12 h at 25–37 °C to form carbamate bonds. The product was precipitated with cold acetone, centrifuged, washed, and vacuum-dried. β-CD (1 *g*) was dispersed in anhydrous DMSO (10 mL) and activated with 1,1′-carbonyldiimidazole (0.9 g) under stirring for 4 h at room temperature to form reactive carbonate groups. Hepcidin (1 mg, dissolved in DMSO) was then introduced, and the reaction continued for 12 h at 25–37 °C, allowing carbamate bond formation between β-CD and hepcidin. The reaction was terminated by adding water, and the product was precipitated with cold acetone, centrifuged, thoroughly washed, and vacuum-dried (Wehl et al., 2025). To confirm the successful conjugation of hepcidin with CMCS and β-CD, Fourier transform infrared spectroscopy (FTIR) was performed. FTIR analysis confirmed the successful conjugation of hepcidin to both CMCS and β-CD. Characteristic spectral changes, including the appearance of amide/carbamate-related bands and shifts in the hydroxyl and amino group vibrations, verified successful bond formation without altering the chemical structure of the carrier materials.

### 2.2 Diets

A basal diet was formulated for common carp according to their nutritional requirements (Table 1) and analyzed for proximate composition following AOAC (1998) procedures. Four experimental diets were prepared: a control diet (basal diet only), a diet supplemented with synthetic hepcidin, a diet containing CMCS-encapsulated hepcidin, and a diet containing β-CD-conjugated hepcidin. Hepcidin was incorporated into the respective diets at 50 mg/kg diet (0.05 g/kg). This inclusion level was selected based on previous study demonstrating the immunomodulatory and protective effects of dietary hepcidin in fish at doses 30 and 60 mg/kg diet (Chen et al., 2020).

**Table 1:** Ingredients and chemical composition of the basal diet.

Ingredients	g/KG	Proximate composition	Percentage
Fishmeal^1^	120	Crude protein	38.14
Meat meal^2^	240	Crude lipid	6.57
Soybean meal	234	Dry matter	90.12
Wheat meal	300	Ash	6.44
Fish oil	7	Energy (kcal/kg)	4088.27
Soybean oil	10		
Corn flour	70		
L-Lysine^3^	7		
L-Methionine^3^	7		
Vitamin premix^a^	2.5		
Mineral premix^b^	2.5		

1- Pars kilka Co., Mazandaran, Iran (Kilka powder analysis; Protein: 70–72%, Fat: 8–11%, Ash: 11.6%, Moisture: 7–9%). 2- Makianmehr Co., Golestan, Iran. 3- Morghenojan Co., Tehran, Iran.

a Vitamin premix (per kg of diet): vitamin A, 2000 IU; vitamin B_1_ (thiamin), 5 mg; vitamin B_2_ (riboflavin), 5 mg; vitamin B_6_, 5 mg; vitamin B_12_, 0.025 mg; vitamin D_3_, 1200 IU; vitamin E, 63 mg; vitamin K_3_, 2.5 mg; folic acid, 1.3 mg; biotin, 0.05 mg; pantothenic acid calcium, 20 mg; inositol, 60 mg; ascorbic acid (35%), 110 mg; niacinamide, 25 mg.

b Mineral premix (per kg of diet): MnSO_4_, 10 mg; MgSO_4_, 10 mg; KCl, 95 mg; NaCl, 165 mg; ZnSO_4_, 20 mg; KI, 1 mg; CuSO_4_, 12.5 mg; FeSO_4_, 105 mg; Co, 1.5 mg.

All dry ingredients were thoroughly mixed before adding the appropriate hepcidin preparation and sufficient water to obtain a homogeneous dough. The dough was pelleted using a meat grinder, dried at 30 °C for 24 h, and stored at −20 °C until use.

### 2.3 Experimental condition

This study was conducted over 8 weeks at the Faculty of Natural Resources, Gonbad Kavous University. A total of 360 common carp (*Cyprinus carpio*) fingerlings, sourced from a local farm (Gulestan, Iran) and with an average initial weight of 5.05 ± 0.22 g (mean ± SE), were used. Before the experiment, holding tanks were thoroughly cleaned and disinfected using salt, followed by rinsing and filling with dechlorinated water (prepared by storing municipal water for 24 h). Fish were acclimated to the laboratory conditions for two weeks in fiberglass tanks (2000 L), during which they were fed the basal control diet (Faradaneh, Shahrekord, Iran). Subsequently, the fish were randomly allocated to 12 tanks, each with a capacity of 200 L, resulting in a stocking density of 30 fish per tank. These tanks were grouped into 4 treatment categories, with 3 replicate tanks per treatment. The experimental diets consisted of: a control diet lacking hepcidin (CTR), the basal diet supplemented with 0.05 g/kg synthetic hepcidin (Hep), the basal diet supplemented with 0.05 g/kg nano-CMCS encapsulated hepcidin (CMCS-Hep), and the basal diet supplemented with 0.05 g/kg nano- β-CD encapsulated hepcidin (βCD-Hep). The fish were fed these diets for a duration of 8 weeks. Each tank was equipped with an airstone to maintain adequate dissolved oxygen levels. Water, sourced from the municipal supply, was continuously dechlorinated (24–48 h of storage) before use. Daily water exchange was performed before feeding, and any uneaten feed was promptly removed via siphoning. Feeding occurred three times per day (8:00 and 12:00 AM, 4:00 PM), with ration sizes adjusted according to the fish’s actual feed intake. Throughout the experimental period, crucial water quality parameters were monitored and maintained within the following ranges: temperature at 22.8 ± 0.2 °C, dissolved oxygen at 6.74 ± 0.02 mg/L, and pH at 7.4 ± 0.1. The photoperiod was 12 h of light and 12 h of darkness. At the end of the 8-week feeding trial, and immediately before the *A. hydrophila* challenge, all fish within each tank were weighed. The fish were then anesthetized using clove powder (140 mg/L).

### 2.4 Growth parameters

The body weight of each fish was measured at the commencement of the experiment and again after the completion of the eight-week feeding trial. Growth performance in common carp was subsequently assessed by calculating the following growth indices using standard equations (Khanzadeh et al., 2025).

$$\text{Weight}\;\text{Gain}\;\left(\%\right)\;=\frac{Final\;Weight\;\left(g\right)\;-\;Initial\;Weight\;\left(g\right)}{Initial\;Weight}\;\times \;100$$

$$Specific\;Growth\;Rate\;\left(\%\;day\right)\;=\frac{\left[Ln\;\left(Final\;Weight\right)-\;Ln\;\left(Initial\;Weight\right)\right]}{\;\text{Days}}\times \;100$$

$$\mathrm{Feed}\;\mathrm{Conversion}\;\mathrm{Ratio}\;\left(g\;/\;g\right)=\frac{\mathrm{Feed}\;\mathrm{Consumed}}{\mathrm{Weight}\;\mathrm{Gain}}$$

$$Survival\;Rate\;\left(\%\right)\;=\frac{Number\;of\;Survived\;Individuals}{Initial\;Number\;of\;Individuals}\times \;100$$

### 2.5 *A. hydrophila* challenge

To determine the LD50 of *A. hydrophila*, serial 10-fold dilutions ranging from 10^5^ to 10^8^ CFU/mL were prepared in PBS. Then, 0.1 mL of each dilution was administered intraperitoneally to groups of 10 fish (30 fish per treatment). Mortality was recorded daily for 10 days post-injection, and the LD50 was estimated by probit analysis using specialized statistical software. On the basis of this preliminary determination, fish in the main challenge trial were injected intraperitoneally with 0.1 mL of bacterial suspension standardized to the LD50 dose of 1 × 10^8^ CFU/mL for common carp (Mazandarani et al., 2025).

### 2.6 Sampling and measurements

At the end of the 8-week feeding trial (before the challenge test) and after the 14-day challenge with *A. hydrophila*, 24 h of feed deprivation occurred. Following these periods, fish were anesthetized using clove powder (140 mg/L, Hajirezaee et al., 2024) to facilitate blood collection. Blood samples were aseptically drawn from two fish per replicate tank (six fish per treatment group) via the caudal vein using heparinized syringes. Immediately following collection, blood samples were transferred into vials and maintained at 4 °C. For serum isolation, blood samples underwent centrifugation at 3000 rpm for 10 min in a centrifuge (Khanzadeh et al., 2025). The sera were carefully collected and stored at −20 °C for future analysis of glucose, cortisol, complement component 3 (C3), and complement component 4 (C4). Immediately after blood sampling, liver tissues were excised from the same 2 fish per replicate tank (6 fish per treatment group). These liver samples were promptly processed for the determination of hepatic enzymes including alanine aminotransferase (ALT), aspartate aminotransferase (AST), alkaline phosphatase (ALP), and lactate dehydrogenase (LDH) by diagnostic reagent kits procured from Pars Azmon, Iran.

### 2.7 Statistical analysis

Statistical analyses were conducted using SPSS software (Version 21.0; IBM Corp., Armonk, NY, USA). Graphs were drawn using GraphPad Prism version 10.6.1 (GraphPad Software, San Diego, CA, USA). Before analysis, the normality of the data was assessed using the Shapiro-Wilk test. Subsequently, the homogeneity of variances across the different experimental groups was evaluated via Levene’s test. Growth performance was analyzed by one-way Analysis of Variance (ANOVA). For parameters measured before and after the bacterial challenge, a two-way Analysis of Variance (ANOVA) was employed. Where significant differences were detected by ANOVA, Tukey’s post hoc test was performed with a significance level set at *P* < 0.05. This test was used to pinpoint specific pairs of groups that exhibited statistically significant differences in their means. All quantitative data are presented as the mean ± standard error (SE). Spearman’s rank correlation analysis was performed to evaluate the strength and direction of associations among growth performance parameters. All statistical analyses were performed using tank means as the experimental observations, because dietary treatments were assigned at the tank level; therefore, each tank represented one biological replicate and the individual fish sampled from each tank served as subsamples.

## 3 Results

### 3.1 Growth performance

During this experiment, no mortality was recorded in any of the treated groups. Fish administered with CMCS-Hep and βCD-Hep had significantly higher final weight when compared to the CTR group (*P* ˂ 0.05). Comparing all treated groups, CMCS-Hep-treated carp showed higher final weight in comparison with Hep-treated fish (*P* ˂ 0.05). Conversely, no significant alterations were indicated in the weight gain, weight gain percent, feed conversion ratio, and specific growth rate (*P* > 0.05, Table 2).

**Table 2:** Growth performance in common carp (*C. carpio*) fed with unsupplemented (CTR), 0.05 g/kg synthetic hepcidin (Hep), 0.05 g/kg carboxymethyl chitosan nano hepcidin (CMCS-Hep), and 0.05 g/kg cyclodextrin nano hepcidin (βCD-Hep) for 8 weeks.

Experiment groups	Parameters						
	IW (g)	FW (g)	WG (g)	WG (%)	FCR	SGR (%/day)	Survival rate (%)
CTR	4.78^a^	11.15^a^	6.37^a^	137.61^a^	1.66^a^	1.51^a^	100±0.00
Hep	4.95^a^	11.59^ab^	6.63^a^	142.13^a^	1.61^a^	1.53^a^	100±0.00
CMCS-Hep	5.25^a^	13.02^c^	7.77^a^	151.14^a^	1.33^a^	1.62^a^	100±0.00
βCD-Hep	5.21^a^	12.41^bc^	7.19^a^	143.86^a^	1.45^a^	1.55^a^	100±0.00
P-value ANOVA	*P* = 0.2741	*P* = 0.0010	*P* = 0.0843	*P* = 0.8901	*P* = 0.0572	*P* = 0.8484	0
SEM	0.193	0.335	0.408	12.32	0.092	0.089	0

Initial weight (IW), final weight (FW), weight gain (WG), weight gain (WG%), feed conversion ratio (FCR), Specific growth rate (SGR). Different lowercase letters mean significant differences between different diets. Different letters designate significant differences as determined by one-way ANOVA and Tukey’s test. (Mean ± SEM, n = 15).

**Fig. 2:**
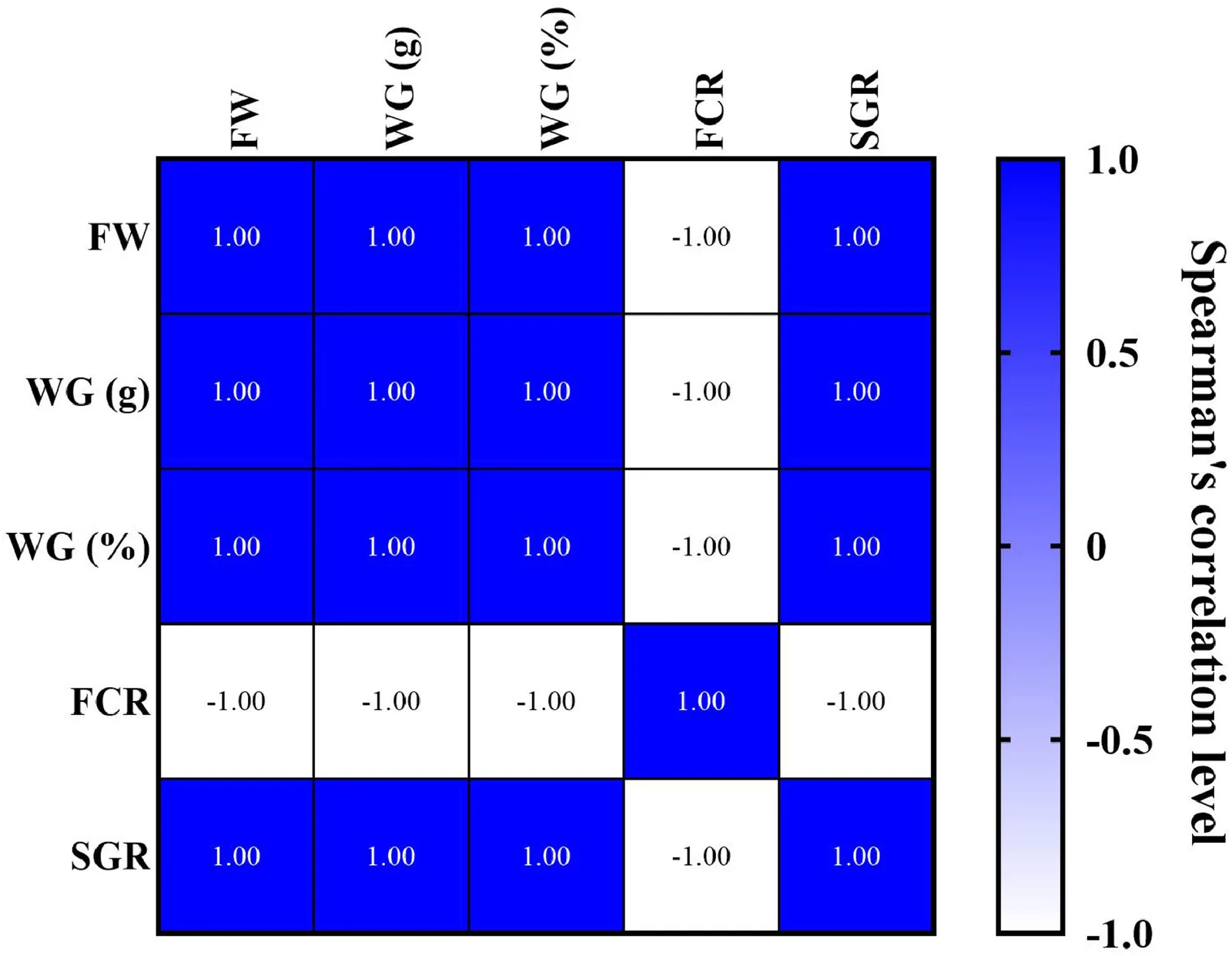
Spearman’s correlation matrix among growth performance parameters in the experimental groups. Spearman’s rank correlation analysis was performed to evaluate the strength and direction of associations among growth performance parameters. Correlation coefficients were calculated and visualized as a heatmap, with positive values indicating direct relationships and negative values indicating inverse relationships.

The correlation analysis showed a strong positive association among FW, WG (g), WG (%), and SGR, indicating that these growth-related parameters increased together. In contrast, FCR was strongly and negatively correlated with FW, WG (g), WG (%), and SGR, suggesting that better growth performance was associated with a lower FCR. Overall, the results indicate a clear positive relationship among growth traits, whereas FCR showed an inverse relationship with the growth indices (Fig. 2).

### 3.2 Biochemical factors

Liver-related enzyme activity for ALT, AST, ALP, and LDH was affected by both diet and challenge test. Before the challenge trial, ALT, AST, and LDH showed significant enhancement in all treatments compared to the CTR group (*P* ˂ 0.0001, Table 3). There was no significant difference in ALP between different treatments before the challenge test (*P* = 0.198, Table 3). After the challenge trial, ALT, AST, ALP, and LDH followed the same pattern with enhancement in all treated groups in comparison with the CTR group (*P* ˂ 0.0001, Table 3). The effect of time was significant in all treated groups for the measured parameters (ALT, AST, ALP, and LDH; *P* < 0.05); however, the interaction between treatment and time was not significant for any parameter (Table 3, *P* > 0.05).

**Table 3:** Biochemical factors in common carp (*C. carpio*) fed with unsupplemented (CTR), 0.05 g/kg synthetic hepcidin (Hep), 0.05 g/kg carboxymethyl chitosan nano hepcidin (CMCS-Hep), and 0.05 g/kg cyclodextrin nano hepcidin (βCD-Hep) for 8 weeks and before and after challenge with *A. hydrophila*.

Experiment groups	Parameters			
	ALT (u/mg protein)	AST (u/mg protein)	ALP (u/mg protein)	LDH (u/mg protein)
Before Challenge				
CTR	27.08^b^	154.50^b^	13.45^a^	139.17^b^
Hep	22.80^a^	139.83^a^	12.56^a^	120.83^a^
CMCS-Hep	19.93^a^	137.83^a^	11.50^a^	110.33^a^
βCD-Hep	20.81^a^	128.00^a^	11.68^a^	109.00^a^
After Challenge				
CTR	32.48^C^	173.33^B^	16.00^B^	151.83^B^
Hep	26.20^B^	152.50^A^	14.40^AB^	136.37^AB^
CMCS-Hep	20.51^A^	143.33^A^	13.20^A^	121.17^A^
βCD-Hep	22.43^AB^	142.30^A^	13.85^A^	125.83^A^
Two-way ANOVA				
Diets	*P* < 0.0001	*P* < 0.0001	*P* = 0.0047	*P* < 0.0001
Challenge (Time)	*P* = 0.0016	*P* = 0.0002	*P* = 0.0002	*P* < 0.0001
Diet × Challenge	*P* = 0.1522	*P* = 0.4256	*P* = 0.9092	*P* = 0.8650
SEM	1.06	3.97	0.63	3.98

Different lowercase letters (a, b, c) within the same column indicate significant differences among dietary treatments before, and different uppercase letters (A, B, C) within the same column indicate significant differences among dietary treatments after bacterial challenge. Different letters designate significant differences as determined by Tukey’s test. (Mean ± SEM, n = 6).

### 3.3 Stress and immune factors

**Fig. 3:**
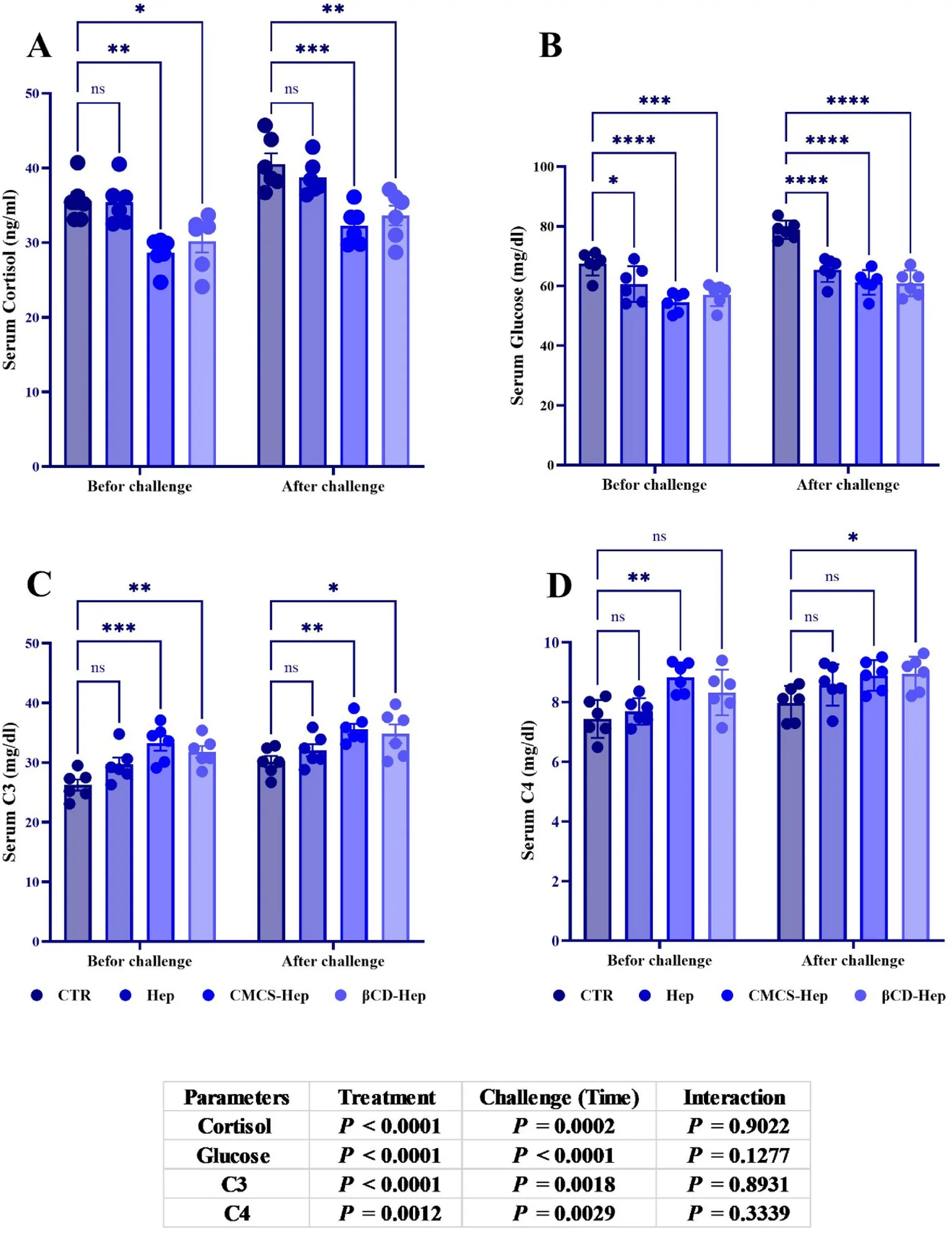
**A-D.** Stress (Cortisol, A; Glucose, B) and immune parameters (Complement component 3, C; Complement component 4; D) in common carp (*C. carpio*) fed with un-supplemented (CTR), 0.05 g/kg synthetic hepcidin (Hep), 0.05 g/kg carboxymethyl chitosan nano hepcidin (CMCS-Hep), and, 0.05 g/kg cyclodextrin nano hepcidin (βCD-Hep) for 8 weeks and after and before challenge with *A. hydrophila*. Different lowercase letters (a, b, and c) mean significant differences between different diets before bacterial challenge. Different uppercase letters (A, B, and C) indicate significant differences between different diets after bacterial challenge. Different letters designate significant differences as determined by Tukey’s test. (Mean ± SEM, n = 6). ns: *P* ≥ 0.05, *: *P* ≤ 0.05, **: *P* ≤ 0.01, ***: *P* ≤ 0.001, ****: *P* ≤ 0.0001.

In parallel with liver biochemical factors, stress and immune indices in common carp serum were influenced by diet and challenge experiment. Before and after challenge with *A. hydrophila*, cortisol levels were lower in fish fed CMCS-Hep and βCD-Hep diets than in those fed CTR and Hep diets (*P* < 0.0001). For glucose levels, all fish groups fed diets administered with Hep, CMCS-Hep, and βCD-Hep demonstrated a significant decrease in their serum compared to the CTR group before and after the challenge test (*P* < 0.0001). Moreover, common carp fed diets containing CMCS-Hep and βCD-Hep exhibited an enhancement in C3 level relative to the CTR group both before and after the challenge trial (*P* < 0.0001). Before the challenge test, the highest C4 level was measured in fish fed the CMCS-Hep diet, whereas after challenge, a significant increase was seen in the C4 level in the common carp serum fed CMCS-Hep and βCD-Hep diets (*P* = 0.0012; Fig. 3). The effect of time was significant in all treated groups for the measured parameters (cortisol, glucose, and serum C3 and C4; *P* < 0.05); however, the interaction between treatment and time was not significant for any parameter (Fig. 3, *P* > 0.05).

## 4 Discussion

The emergence of AMPs like hepcidin as potential therapeutic agents has opened new avenues for aquaculture research (Shabir et al., 2018; Naiel et al., 2023; Das et al., 2026). However, natural AMPs show instability and limited oral bioavailability, restricting their clinical applications (Divyashree et al., 2020; Del Genio et al., 2022). The approach of AMPs encapsulation within nanomaterials improves the efficacy and stability of these biomolecules via not only reducing toxicity to host cells but also combating infections induced by drug-resistant bacteria (Zheng et al., 2025). Therefore, this investigation was designed to study the efficacy of nanoencapsulated hepcidin within nano-CMCS and nano-βCD compared to natural hepcidin on common carp growth, liver-related enzymes, and some stress and immune indices. Results demonstrated that nanoencapsulated hepcidin, especially within chitosan, could produce marked effects on fish growth and physiological indices. The better performance of CMCS over β-CD is likely attributable to its mucoadhesive nature and positive surface charge, which enhance interaction with the intestinal mucus layer, prolong gastrointestinal residence time, and improve the stability and uptake of encapsulated hepcidin. In contrast, although β-CD enhances peptide stability through inclusion complex formation, its lower mucoadhesion may limit intestinal retention and absorption. These differences may explain the greater physiological and immunological benefits observed with CMCS-encapsulated hepcidin in common carp.

In this experiment, the addition of natural hepcidin to the feed could not improve the growth performance of common carp, whereas encapsulated hepcidin within nanoparticles, especially nano-CMCS, could enhance the final weight in treated fish. Even though previous scholars demonstrated that various antimicrobial peptides could significantly increase growth performance, digestive enzyme activities as well as gut microbiota communities in cultured fish (Liu et al., 2020; Wang et al., 2021; Zhou et al., 2022), it seems that as a result of the completely minute size, these nanoparticles are capable of penetrating through biological barriers in vivo and enhancing hepcidin efficacy and stability. Liver-specific enzymes, e.g., ALT, AST, ALP, and LDH, are sensitive indices of liver histopathological changes. In the present study, following the administration of hepcidin and encapsulated hepcidin, lower activity of these enzymes was noted. Therefore, it can be assumed that these additives might be safe enough, even though further toxicological studies are warranted to prove it.

In this study, exposure of common carp to *A. hydrophila* altered some biochemical and immunological traits, including stress indices cortisol and glucose, demonstrating potent stress responses. Conversely, nano-CMCS and nano-βCD encapsulated hepcidin could alleviate the stress responses, resulting in lower levels of cortisol and glucose in carp serum. Similarly, nano chitosan gel could ameliorate the stress biomarkers in the *Candida albicans*-challenged Nile tilapia (*Oreochromis niloticus*) (Mahboub et al., 2025). The authors proposed that the small-sized nanochitosan could diminish the fungal agents via antimicrobial activity and gradually relieve the potent stress condition (Mahboub et al., 2025). The fish complement system, composed of nearly 35 proteins, is present in serum, tissue fluid, as well as cell membrane surface. This system has a vital role in fish defence by involving non-specific and adaptive immunity. Three distinct pathways of complement activation include the classical route, lectin route, and alternative route (Wu et al., 2022). In the classical route activation, the active C1s cleaves C4 and C2 complement components, producing C4b and C2b, respectively. C4b binds covalently to a target surface via its intramolecular thioester site, while C2b, with serine protease activity, interacts with C4b, forming a complex, C4b2b, to cleave C3, the main complement component, producing C3a and C3b fragments with some physiological activities including opsonization and anaphylatoxic leukocyte activation (Nehlah et al., 2023). In the current study, enhancement of C3 and C4 levels was recorded following the administration of nano-encapsulated hepcidin. Notably, encapsulated hepcidin revealed better performance than natural hepcidin. It was previously suggested that the presence of nanoparticles for a more extended period in the bloodstream might enhance the bioavailability of these AMPs. Chitosan nanoparticles are also efficiently taken up by phagocytic cells to affect the systemic immune system compared to natural chitosan (Khani Oushani et al., 2020).

## 5 Conclusion

In conclusion, this study indicated that administering nano-CMCS-encapsulated hepcidin exhibited a significantly better protective effect than feeding natural hepcidin alone in terms of final growth, liver enzymes, stress, and immunological factors in common carp. However, some challenges, e.g., fish host safety, regulatory approval, and long-term efficacy, should be addressed to highlight encapsulated AMPs' therapeutic applications. A practical consideration for the application of hepcidin in aquaculture is its production cost. In the present study, a highly purified commercial synthetic hepcidin was used to ensure reproducibility and to accurately evaluate its biological effects. However, the high cost of synthetic peptide production and the use of 99% pure synthetic hepcidin may limit its direct large-scale application in commercial aquaculture. In this regard, nanoencapsulation may improve the cost-effectiveness of hepcidin by enhancing its stability and oral bioavailability, thereby reducing peptide loss and potentially lowering the effective dose required. Moreover, future studies should evaluate alternative and more economical production strategies, such as recombinant expression systems or peptide engineering, together with comprehensive cost–benefit analyses before commercial implementation. This study has some limitations, including the evaluation of a single dietary dose and one fish species. Further studies using different doses, species, and long-term safety assessments are needed to confirm the broader applicability and safety of nanoencapsulated hepcidin in aquaculture.

## Ethical approval

All experiments were conducted in accordance with relevant guidelines and regulations. The experiment was conducted in compliance with the ARRIVE (Animal Research: Reporting of In Vivo Experiments) guidelines. The experiment was conducted following the ethics and animal care committee of the faculty of sciences of the University of Tehran (357; 8 November 2000).

## Funding

This work is based upon research funded by the Iran National Science Foundation (INSF) under project No.4039,304.

## Competing interests

Authors declare no conflict of interest**.**

## Availability of data and material

The data and materials that support the findings of this study are available from the corresponding author upon reasonable request.

## Consent for publication and participation

The authors declare that they have consent for publication and participation.

## AI statement

Artificial intelligence methods were not utilized in this research.

## Ethics declaration

This study was conducted in accordance with the ARRIVE (Animal Research: Reporting of In Vivo Experiments) guidelines. This study was approved by the University of Tehran. (Approval No. 357; 8 November 2000)

## CRediT authorship contribution statement

**Ehsan Ahmadifar:** Writing – review & editing, Writing – original draft, Visualization, Validation, Supervision, Project administration, Methodology, Investigation, Funding acquisition, Conceptualization. **Sedigheh Mohammadzadeh:** Writing – review & editing, Writing – original draft, Visualization, Methodology, Data curation, Conceptualization. **Mohsen Shahriari Moghadam:** Writing – review & editing, Writing – original draft, Validation, Methodology, Investigation. **Mostafa khajeh:** Writing – review & editing, Writing – original draft, Visualization, Methodology. **Hossein Adineh:** Writing – review & editing, Writing – original draft, Methodology, Investigation. **Sevdan Yilmaz:** Writing – review & editing, Writing – original draft, Visualization, Validation, Supervision. **Najmeh Sheikhzadeh:** Writing – review & editing, Writing – original draft, Visualization, Validation, Supervision. **Majid Khanzadeh:** Writing – review & editing, Writing – original draft, Software, Formal analysis.

## Declaration of competing interest

The authors declare that they have no known competing financial interests or personal relationships that could have appeared to influence the work reported in this paper.
